# Trust and the regulation of pharmaceuticals: South Asia in a globalised world

**DOI:** 10.1186/1744-8603-7-10

**Published:** 2011-04-29

**Authors:** Petra Brhlikova, Ian Harper, Roger Jeffery, Nabin Rawal, Madhusudhan Subedi, MR Santhosh

**Affiliations:** 1 School of Social and Political Science, University of Edinburgh, Edinburgh, UK; 2 Department of Social Anthropology, Tribhuvan University, Kathmandu, Nepal; 3 Centre for Trade and Development, New Delhi, India

## Abstract

**Background:**

Building appropriate levels of trust in pharmaceuticals is a painstaking and challenging task, involving participants from different spheres of life, including producers, distributors, retailers, prescribers, patients and the mass media. Increasingly, however, trust is not just a national matter, but involves cross-border flows of knowledge, threats and promises.

**Methods:**

Data for this paper comes from the project 'Tracing Pharmaceuticals in South Asia', which used ethnographic fieldwork and qualitative interviews to compared the trajectories of three pharmaceuticals (Rifampicin, Oxytocin and Fluoxetine) from producer to patient in three sites (north India, West Bengal and Nepal) between 2005-08.

**Results:**

We argue that issues of trust are crucial in reducing the likelihood of appropriate use of medicines. Unlike earlier discussions of trust, we suggest that trust contexts beyond the patient-practitioner relationship are important. We illustrate these arguments through three case studies: (i) a conflict over ethics in Nepal, involving a suggested revised ethical code for retailers, medical representatives, producers and prescribers; (ii) disputes over counterfeit, fake, substandard and spurious medicines, and quality standards in Indian generic companies, looking particularly at the role played by the US FDA; and (iii) the implications of lack of trust in the DOTS programmes in India and Nepal for the relationships among patients, government and the private sector.

**Conclusions:**

We conclude that the building of trust is a necessary but always vulnerable and contingent process. While it might be desirable to outline steps that can be taken to build trust, the range of conflicting interests in the pharmaceutical field make feasible solutions hard to implement.

## Background

In conducting our research tracing three pharmaceuticals (Rifampicin, Fluoxetine and Oxytocin) from production to consumption in South Asia we became increasingly interested in participants' concern with 'trust' as a key feature of how different stakeholders related to the pharmaceuticals industry. We turned our attention to how a lack of trust was a key problem in ensuring that these drugs were appropriately produced, stored, distributed, prescribed and consumed. We understand trust 'in rational or calculative terms, as a form of confidence based on incentives, rules, or institutional features that gives one person reason to believe that another person will protect his or her interests' [[1]: 1133]. Most analyses of trust in the health sphere focus on the dyadic practitioner-patient relationship and generalised trust of patients in health care providers and systems (see [2] for a review). This approach has a long history: much of the classic literature around the 'profession of medicine' - for example the work by Talcott Parsons - explores why doctors inspire trust (in his view, because they have the imprimatur of science, long training producing an *esprit de corps*, and an ethical code that protects patients) [3]. Arrow pointed to the placebo effect:

It is a commonplace that the physician-patient relation affects the quality of the medical care product.... That purely psychic interactions between physician and patient have effects which are objectively indistinguishable in kind from the effects of medication is evidenced by the use of the placebo as a control in medical experimentation [[4]: 951].

Note two aspects of this approach:

a. a focus on individual patients and practitioners, with little attention paid to the wider context;

b. a generalisation from the physicians' viewpoint, and their claim of the benefits to patients if they trust physicians.

The argument has been extended to consider a physician's trust in a patient in the context of drugs with a potential for abuse (e.g., [5]). However, interpersonal trust in practitioner-patient relationship is also dependent on the availability and quality of health services [2]. Gilson [6] follows Giddens [7] in noting that although complex public health systems require generalised or disembedded trust (trust in complex social systems and institutions following sets of rules, norms, laws and customs) these trust relationships are also 'rooted in the inter-personal relationships that regularly affirm its affective bases. In other words, the micro and the macro-levels of trust are interconnected' [[6]: 361]. Because 'systems' are disembedded from local contexts and personal relations [8] and because self-interested individuals might be tempted to misrepresent and lie despite the rules and norms [[9]: 389, referring to [10]], ensuring sufficient trust presents a challenge. Many of these arguments apply, *mutatis mutandis*, in India and in Nepal. But here, as in some other parts of the world, physicians face additional complications in sustaining the trust of their patients, because there are state-sanctioned competitors - trained and practising Ayurveda, Unani, Siddha or combinations of these with so-called 'allopathic' medicine - outside the control of the professional associations and regulatory bodies that deal with cosmopolitan medicine [11,12].

**Figure 1:**
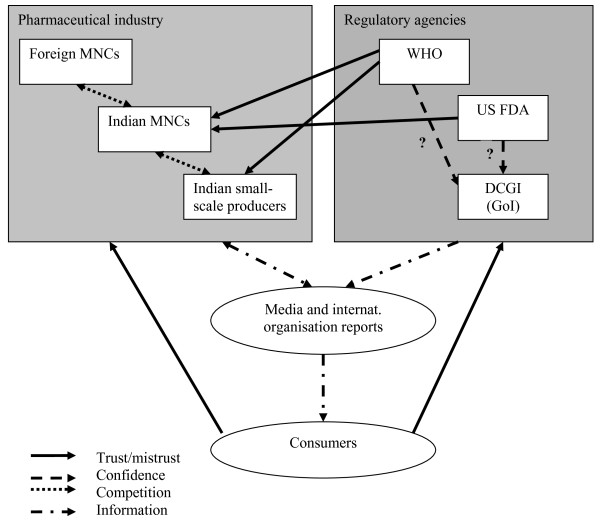
Key relationships affecting trust in pharmaceutical products in India

Trust can be built when others - not directly involved in a relationship or situation - attest to the trustworthiness of actors or groups of actors, as well as when actors themselves behave in ways that appear to be disinterested. But Mechanic lists several reasons for a declining trend of trust in medical institutions in the US and also in the UK: the influence of television and other media on public opinion, the fragmentation of community, the widespread dissemination of information on political and other violations of public trust, the restructuring of the economy, and the increase in health care provision by for-profit institutions, which present medicine as a marketplace and view patients as consumers [13]. Many of these processes are also visible in India and Nepal, but they impinge upon a system that started with less public confidence and greater reasons for mistrust. But there is a lacuna in this literature, relating to the need to consider the fact that, increasingly, disembedded systems cross national boundaries and resemble global assemblages 'through which global forms of techno-science, economic rationalism, and other expert systems gain significance' [[14]: 3]. How these global processes impact on local trust relationships is one key element of this paper (see Figure 1 for a graphical representation of these relationships).

Trust is fragile because negative events are more visible, they carry greater psychological weight, and they are perceived to be more credible [[12], referring to [15]]. Reporting of negative events raises doubts and feelings of insecurity about the incentives and behaviour of medical practitioners. Furthermore, the reporting of scandals but not 'good practice' gives the impression that untrustworthy behaviour is normal and pervasive [16]. Similar issues of trust arise with respect to the regulatory framework that is supposed to ensure that new drugs are safe and efficacious, and that effective pharmacovigilance will alert patients and practitioners to new health threats. There is a danger of 'industry capture,' if 'the pharmaceutical industry influences the perspective of the regulatory agency-so it comes to adopt their interests over and above those of patients' [[17]: 1498]. But if the industry is not seen as a unitary force, but rather one riven by conflicting interests as well as (on occasion) brought together in support of the industry as a whole, we need to ask which sectors of the industry are most successful in gaining regulatory support. In focusing on trust relationships involved in the regulatory frameworks that affect all sectors of pharmaceutical supply chains - frameworks and agencies that are supposed to ensure that all medicines are safe, effective and produced to specified quality standards, and to enforce standards for drug distribution, marketing and promotion - we note the potential for conflict as well as co-operation amongst these agencies and institutions, rather than focusing just on those dealing with the approval of new chemical entities.

**Figure 2:**
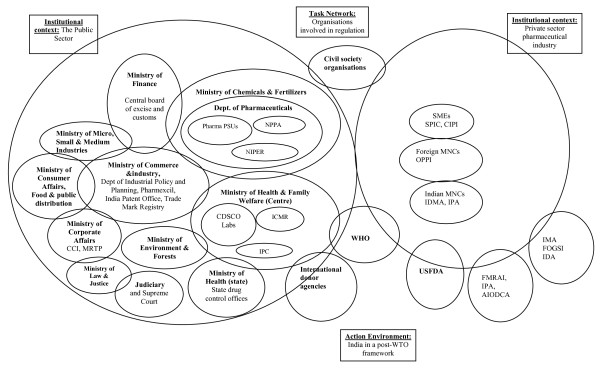
**The organizations involved in pharmaceutical regulation in India, and their contexts, adapted from **[60]

Trust, then, is a much more general issue in health systems and in pharmaceuticals supply chains in particular than has so far been addressed. Although there is a bewildering array of stakeholders concerned with pharmaceuticals in any country (Figure 2 represents those we have identified in India, for example) we focus on those most central to our interests. We should note that, for most retailers and wholesalers we spoke to, the specific significance of pharmaceuticals - that they are dangerous if misused or kept, handled or distributed without attention to the appropriate standards - are not of key concern for them as businessmen and women.

### The pharmaceuticals market in South Asia

The Indian pharmaceuticals market is growing fast, and it is set in one of the most complex health systems in the global South - ranging from '5-star' hospitals offering cutting edge surgery to medical tourists as well as the local elite, to run-down clinics and health centres that function barely at all, and are surrounded by unqualified practitioners who prescribe a full range of treatments, often from several systems of medicine. India is an increasingly significant pharmaceuticals market, although the vast majority of pharmaceuticals available in India are already off patent, and generics are likely to dominate the market for the foreseeable future [18]. By contrast, Nepal has a small and struggling production sector, and elite provision is truncated. But in both countries, the pharmaceuticals market is saturated with 'branded generics'.

In the simple models popular with industry analysts, the Indian and Nepal drug distribution systems have only four or five layers: pharmaceutical manufacturers; clearing (or carrying) and forwarding agents [CFAs]/depots/super stockists; stockists; wholesalers; and retailers. These models also define only a small number of routes through which drugs flow. The main difference for Nepal is the absence of the layer of CFAs, super stockists or depots.

On closer analysis, this neat picture breaks down. Estimates vary dramatically as to the numbers within each of these categories, suggesting that the boundaries are not clear-cut. In India, estimates of production companies varies from over 20,000 - widely quoted but rarely substantiated - to 5,000 who are 'active' producers. The numbers of CFAs or super-stockists are rarely given, and numbers seem likely to change quite quickly, since the roles reflect tax and licensing conditions rather than real economic need. Estimates of 60,000 stockists and 500,000 and 600,000 pharmacists in 2000-2005 are based on figures supplied by the All-India Organisation of Chemists and Druggists [19-21]. Industry sources claim that retailers account for about 70-80 per cent of the pharmaceuticals sales in the country, with the remainder being sold directly through hospital pharmacies [22]. In rural and small-town India (accounting for 25-35 per cent of the market) private medical practitioners (whether formally trained or not) often stock most of the medicines they expect to prescribe [23]. Most small hospitals and nursing homes also have in-house pharmacies and many require patients to buy the drugs on the premises, whether they are in- or out-patients. Finally, there are far more who earn a living by prescribing the products of the globalised pharmaceutical industry than the Medical Council of India [MCI] figure of 668,131 for physicians in 2006 [24].

The large-scale companies that produce the active pharmaceutical ingredients are relatively few: most small-scale producers merely produce formulations. But the boundary between the two kinds of companies is not clear-cut. Small companies often act as additional producers for large companies, formulating drugs and packaging them with the name of the large company, on 'loan licences' or contracts that allow them to manufacture a product of another party. In such cases drugs are exactly the same as those produced in factories owned by the large producer. Loan-licensing or sub-contracting may avoid excise duty or sales tax, or allow the large producer to take advantage of the small-scale producer's ability to pay lower wages, with lower social welfare payments and other costs.

The distinction between retailer/pharmacist and practitioner is also often unclear: in much of Nepal and the Indian countryside (and also in smaller towns) patients commonly approach a pharmacist or retailed and receive a diagnosis and medicines (often including powerful prescription drugs) without the intervention of any other kind of practitioner [25]. Similarly, in most small towns and villages, practitioners also dispense the medicines they prescribe without charging a consultation fee. Finally, once medicines have reached a patient, they are not necessarily consumed by that person. Rather, the portion unconsumed may be passed on, sold or traded with other patients; and prescriptions may have a life of their own, generating further drug purchases either for the original patient or for someone else entirely [25,26].

India and Nepal's drugs distribution systems can, then, be described as 'unregulated':

(a) Unlicensed individuals and/or entities trade in drugs that they are not authorized or entitled to deal with or in contravention of the applicable laws, regulations and norms; or (b) Licensed individuals and/or entities trade in drugs that they are not authorized or entitled to deal with or in contravention of the applicable laws, regulations and norms [[27]: 1-2].

This lack of regulation is experienced - to a greater or lesser extent, depending on people's position in the supply chain and their social, economic and cultural capital - as a pharmaceuticals market that must be treated with excessive caution. Individual patients cannot always trust that those who prescribe drugs are qualified to do so (nor that their prescriptions are motivated by the best interests of the patient, rather than the pecuniary interest of the prescriber). Doctors and other prescribers do not feel confident about the quality of many of the companies producing drugs - generic or patented - that they give or sell to their patients. For many years, public sector hospitals and clinics have either supplied no medicines at all, or have provided low cost generic unbranded medicines which have been subject to regular scare stories concerning their efficacy and safety; yet patients have often been asked to pay for these nominally free medicines [23]. Retailers do not always trust companies to supply good quality products at fair prices, and producers do not trust retailers to pay on time or to give their products a fair opportunity in the marketplace. Producers offer retailers financial inducements to substitute their own products for those that may have been prescribed by a doctor or other prescriber: practices like this are well known and further undermine trust in other parts of the system [23,25,28]. Few of the stakeholders trust the regulators who are charged with ensuring that the relevant rules are followed. In all these cases, the local mass media tend to enhance feelings of distrust by publishing horror stories of corruption, counterfeit medicine or of the risks of unqualified practitioners.^1^

## Project Design and methods

'Tracing Pharmaceuticals in South Asia' was designed to integrate the insights of discussions in anthropology of global assemblages and in political economy of global commodity chains; and by integrating 'bottom-up' and 'top-down' approaches to the local and global contexts through specific case-studies.^2 ^We selected three key generic drugs (Oxytocin, Rifampicin and Fluoxetine) on the basis of our prior knowledge of patterns of use in three regions of South Asia (Nepal, West Bengal and Uttar Pradesh). The selected drugs provided contrasting patterns of production, distribution, marketing and retail sales, reflecting, for example, Rifampicin's central role in the Indian and Nepali vertical TB programmes, compared to the more peripheral role of the state in the cases of Oxytocin and Fluoxetine. Our existing knowledge suggested high levels of informal and unregulated use of each of these drugs, which impinge on different global strategies for health, with different priorities for action. While the three drugs provided contrasting insights into many aspects of supply chains, they provided similar information on the role played by trust and mistrust in the overall pharmaceuticals context. Through semi-structured interviews with people throughout the production chain, with representatives of donor agencies, government personnel and some observation of particular settings (such as TB clinics and maternity wards) we established a clear picture of these pharmaceuticals in use. We now turn to three case studies that demonstrate how issues of trust intervene within relationships in the medicine supply chains in India and Nepal. These case studies were selected to illustrate processes at different points in the supply chain, and area examples where we were able to draw upon evidence from a variety of viewpoints to contextualise and triangulate our argument. Whereas in some areas - such as prescribers' trust in particular pharmaceuticals companies and their products - trust relationships are strong and regularly reinforced through gift-giving, social events, and medical representatives' activities, in these cases (and others) trust is threatened, undermined or regularly needs to be renegotiated and re-established.

## Case Study 1

### Responses to a suggested revised ethical code in Nepal

In July 2007 the Government of Nepal released its Guidelines on the Ethical Promotion of Medicines through its Department of Drug Administration [DDA]. While acknowledging that the industry is run as a business, the Director of the DDA noted that this raised ethical issues. In particular concern was raised over costs to patients, and that the giving of 'bonuses' to retailers to stock particular brands had increased^3^; and that substitution of prescribed brands for others has been pushed by producers, driven by the systemic giving of discounts and free samples of medicines.

In response to the release of these guidelines there was widespread protest and resistance from several key organisations representing different positions in the chains of distribution and prescribing (see Table 1). Pharmacists, retailers and wholesalers, medical representatives, producers and the medical profession all responded in ways that eventually made the implementation of the Guidelines impossible. Most respondents bemoaned the lack of consultation over the development of the Guidelines. In a complex institutional network like pharmaceuticals, each set of representatives found reasons to blame others for any identified ethical problem. Sets of vested interests developed responses to the guidelines, making it difficult to know who to trust as accusations and rebuttals were publicised.

**Table 1:** Stakeholders in the conflict over ethical codes for retailers in Nepal

*Organisation*	*Main Objectives*	*Membership*	*Website address*
Department of Drug Administration (DDA)	'To regulate all functions relating drug like misuse and abuse of drugs and its raw materials, to stop false and misleading advertisement and make available safe, efficacious and quality drug to the general public by controlling the production, marketing, distribution, sale, export-import, storage and use of drugs'.	Government department	http://www.dda.gov.np/
Graduate Pharmacists' Association of Nepal (GPAN)	The 'overall development of pharmacy as a profession with the motto, 'Profession of Pharmacy for Better Health Care'.'	'Non-governmental, non-profitable, non-political and fully professional organization of pharmacists'	http://gpan.com.np/
Nepal Chemists and Druggists Association (NCDA)	'Enforcing the price uniformity of drugs within the country'	'Non-governmental organization of pharmaceutical trade professionals' (retailers and wholesalers)	http://www.dda.gov.np/ncda.php
Nepal Medical and Sales Representative Association (NMSRA)	'Establishing state-defined professional values'; 'Eliminating the anti-national practices and domination by foreign companies in labor and business'; and 'helping by all possible means the state mechanism to ensure access to quality health for all'	Trade Union for Medical Representatives	http://nmsra.org.np/client/index.php
Nepal Medical Association (NMA)	'To uplift and preserve the professional standard, values and freedom of the NMA'; 'carry out academic activities...'; and '...improve the health status of the people'.	'Non-governmental professional organisation of the Nepalese medical doctors'	http://www.nma.org.np/
Association of Pharmaceutical Producers of Nepal (APPON)	'Facilitation of Nepalese Pharmaceutical Industries to achieve standard par international & to be Globally Competitive.'	Pharmaceutical companies	http://www.appon.org/

For the Nepal Chemists and Druggist Association [NCDA], the main problem was the attack on the bonus system. The NCDA reacted immediately and lobbied hard for the guidelines to be withdrawn, otherwise retailers and wholesalers would no longer make profits, leaving the companies unscathed. The NCDA argued that the volume of drugs being sold would remain the same, and that the drug companies would never lower prices to compensate for this. In their view, the benefits of stopping bonuses would be transmitted directly, and unfairly, to the producers.

Medical Representatives [MRs] were aggrieved that the guidelines named them as a specific problem as the channel for 'gift' giving. MRs argued that this was because they had no official status and that if they were registered with the DDA, they would be better regulated, and such ethical problems would not arise. For the MRs, the dispute offered an opportunity to escape from a target system that linked their pay levels directly to sales. They argued that the companies pushed them to visit retailers, for example, and this encouraged unethical practices; if they were less accountable to the companies and more to the government of Nepal, they could resist such pressures.

For the Nepal Medical Association [NMA], the problem of bonus giving did not lie with the medical profession, but with retailers and manufacturers. A leading member of the NMA said that if doctors take gifts from companies, responsibility lies with the companies, not the doctors. He argued that doctors trust a company because of their personal experience of the quality of its products, not in return for any gifts they might receive:

'Some doctors might have very good relationship with the specific companies, brand and products. They might trust one specific company based on their own experience and not because of the gift and other benefits. Other people could say that Dr. X is getting advantage from the company Y so that he/she prescribes that specific brand. This might not be true...

The issue of why doctors and pharmacists choose one company's products over another lay behind the responses of many of them to accusations that they did so for their own financial gain. For example, a psychiatrist prescribes the anti-depressant fluoxetine naming the 'multinational' brands (by which he meant Indian branded drugs) rather than Nepali ones so that he can be sure of 'quality'. If a patient fails to respond to his treatment regime, his first thought is to the quality of the compound, rather than to suspect the validity of his original diagnosis. At the main teaching hospital in Kathmandu, psychiatrists are trained to prescribe brand name drugs, rather than writing generic names, with the rationale that in the market place, and with increasing numbers of brands coming and going, this was the best way to be sure of the quality of the drugs. In one hospital a pharmacist in charge of the hospital pharmacy, on discussing the question of trusting the system that procured TB drugs in Nepal, seemed to trust only his own empirical experience of the outcomes of patients, not the government programme nor the international procurement process.

In addition, patients themselves tend to want certain brands, and one pharmacist explained that while he would like to substitute, he cannot, as patients refuse. Another said that in light of increasing competition, and substitution practices, local people trust him rather than the drugs *per se*. A retailer denied that he practised substitution:

'I think that is an immoral practice, and I don't do that here. Our customers trust us and that is why they come here. For my patients, if I do not have a particular brand, we provide them through our friends... I don't give substitutes.'

This retailer blamed the DDA and the government's failure to regulate: but he also rejected the specific proposed guidelines. Blame was regularly laid at the government's door by retailers and by others in the chain. The president of the Association of Pharmaceutical Producers of Nepal linked the gift and bonus giving to the increase in competition in the market. But he defined the problem with the lack of a regulatory framework and the government, rather than with the producers. Another Nepali producer stated that without controlling the situation of bonus giving in India, there was little point in attempting to prevent the issue in Nepal alone.

Not surprisingly, perhaps, none of the participants in the system reflected analytically on what the patterns of gift-giving in the supply chain mean to the participants. In South Asia, as in the USA, there is a 'three-way gift cycle occurring in the medical marketplace between reps, doctors, and patients' that provides a context for building trust in what is otherwise a trust-destroying situation [[29]: 327]. 'The actual everyday pharmaceutical economy is based on social relationships that are forged and strengthened through repetitive and *calculated *acts of giving' [[29]: 332, emphasis in the original]. A key issue, then, in the attempted regulation of drugs in Nepal - made manifest through the release and responses to the ethical guidelines on drug promotion - is that the government has lost its capacity to inspire trust in its regulatory processes. As a result, trust in whether or not a drug works tends to become dependent on individual relationships - between patients and specific retailers, or doctors and particular companies - rather than in the system itself. Different actors are, then, not against regulation *per se*, but ask for different kinds of regulation, depending on their particular definition of why there is an absence of trust.

## Case Study 2

### Disputes over 'counterfeit' medicines and quality control in India

The possibility that a medicine might not be all that it seems is clearly a potential source of mistrust. Patients and prescribers cannot easily tell from a package what it contains, and whether it will fulfil the promise of cure or relief of symptoms: they need to trust the disembedded mechanisms that are designed to assure quality, prevent fraud and obviate the need for further enquiries. But such judgements are not made entirely with reference to a particular packet or pill: they are framed by local, national and international discourses about spurious and counterfeited medicines and the quality standards applied to Indian producers.

Media reports, both globally and within India itself, claim that India is one of the top five sources of counterfeit drugs [30,31].^4 ^Representatives of multinational and large Indian companies regularly put forward accusations that the extent of counterfeiting in India is substantial, dangerous to the public and leading to large losses for legitimate producers. In 2002, a submission from the Confederation of Indian Industry [CII] to the 2003 'Expert Committee on a Comprehensive Examination of Drug Regulatory Issues, Including the Problem of Spurious Drugs', chaired by Dr R A Mashelkar, claimed that the WHO had estimated that

'35% of fake drugs produced in the world come from India, which has a Rs. 4,000 Crore spurious drug market. About 20% of medicines in the country are fake or sub-standard. Of these, 60% do not contain any active ingredient, 19% contain wrong ingredients and 16% have harmful and inappropriate ingredients' [[32]: 76].

But the CII failed to provide the Mashelkar committee with evidence to support its claims, and the WHO denied ever having produced a study with the results attributed to it [[32]: 76-7]. These Indian pharmaceutical companies' unsubstantiated claims seem to be the sole source cited by IMPACT, a body dominated by large pharmaceuticals companies but associated with the WHO [33]. The 2005 European Commission statistics [34] that 75 per cent of the cases of counterfeit medicines seized on the EU borders originated from India has been widely cited [see, e.g., [35]]. By 2007, however, only 35 per cent of medicines seized by the EU and treated as counterfeit came from India, while medicines originating in Switzerland comprised 39 per cent of the total - but this statistic has not been widely cited [36].

According to the PSI the extent of counterfeiting varies dramatically by drug, with Viagra accounting for a sizeable proportion [31]. There is some limited (and not very reliable) evidence for how far this applies in India. A study published in 2007 was based on an attempted random collection of 10,743 samples, of which 23 percent were deemed *prima facie *suspect, but only 8 of these samples (0.3 percent of the original drugs collected) failed an assay test [37].^5 ^A Government-sponsored survey in 2009, based on a model provided by the Indian Statistical Institute, tested 24,136 samples of 61 popular brands from nine therapeutic categories, finding the prevalence of spurious drugs at 0.046 per cent [38]. In several articles, Roger Bate has drawn attention to substandard drug seizures in India, and to his own small-scale studies (in one, of five medicines from 52 pharmacies; in another, a total of 720 samples were purchased). In a response to the Government-sponsored survey mentioned above, he cast scorn on the claim

that its authors had no useful information about areas known for counterfeit drugs. This is inconceivable: New Delhi's Bhagirath Palace and certain markets in Agra and Aligarh are known to me, a foreigner, as major locations of the fake drugs trade [39].

The CII agenda seems to be to draw a clear line between the respectable, safe, large producers and the myriad of small and medium enterprises, and thus to establish trust in the big Indian companies and enhance their export potential. But perhaps, as Delhi's then deputy drug controller said in 2001, 'Fake drugs are not Delhi's problem' and 'a lot of the times it is just old brand rivalry. The big fish cannot bear to find smaller chaps coming out with similar medicines so they say 'spurious, duplicate, &c.' [40]. In other words, the larger companies are trying to generating mistrust in the products of their generic competitors, in order to charge higher prices for their more 'reliable' drugs. This message is supported by leading doctors, as became clear in our interviews. Medical College Professors repeatedly told us that some companies (usually, but not always, the market leader) were the only producers that could be trusted; they pass this attitude onto their students, affecting their prescribing practices after they graduate. In the absence of any pharmacological evidence of differences in the same drugs produced by different producers, we conclude that these processes of inter-company competition lead to many consumers paying more for their drugs than they need to, and for many more to be confused and lacking in faith in the cheaper drugs that they take.

The strategy of the larger producers has helped to generate a climate in which their own claims to be above suspicion have also been challenged. India has the highest number of production facilities approved by the US Food and Drugs Administration [FDA] outside the US. In 2001, Schedule M of the Drugs and Cosmetic Act, 1940 (specifying requirements on manufacturing facilities of allopathic drug producers) was amended to comply with FDA and WHO current Good Manufacturing Practice [cGMP] standards and was mandatory by 2003. Large companies complied, but some smaller companies that focus on domestic markets were unable to reach the new standards. They are being squeezed out by the combination of the workings of the trade-related aspects of intellectual property rights [TRIPS] within the World Trade Organisation [WTO], intensified competition and these more stringent cGMP standards. Of the 5877 manufacturing units with drug manufacturing licenses in 2002, only around 400 complied with WHO cGMP requirements, of which 300 were large scale units [32].

Despite the adoption of stricter production standards the credibility of Indian drug regulatory authority is being challenged by US and European regulators. In 2008 WHO threatened to de-recognise Indian national regulatory authorities for failing to impose cGMP standards at three public vaccines producers. Such de-recognition would have had severe implications for Indian exports. Thus, like other developing countries with weak regulatory capacities struggling with low capacity [41], India faces a trade-off between the implementation of stricter rules and viability of local production (at least in the context of small-scale producers). Indian regulators are ambivalent: coming down hard on smaller producers might (after a lag) raise the trustworthiness of all Indian producers abroad, but at the cost of considerable disruption of supplies to the domestic market, and a rise in prices. Similarly, the larger companies face a dilemma: to portray others as producers of counterfeit drugs may weaken the position of smaller producers internally, but damage the overall image of India as a drug producing country. The outcome of these contradictory pressures is delays in the strict enforcement of the specified rules.

Confidence in the FDA's regulatory processes was questioned in 2008, when 62 deaths were associated with Baxter's heparin products in the US. Baxter imported contaminated heparin from China, from a company that was not cGMP certified. The same year a report revealed important shortcomings in FDA records about importers and about inspections visits of their facilities as well as a low number of inspections carried out abroad that are not repeated on a regular basis [42]. To improve oversight of foreign facilities in two key source-countries of pharmaceuticals, FDA offices were established in India (in January 2009) [43] and in China.

Before the India office was opened, Ranbaxy's Dewas and Paonta Sahib plants were inspected by the FDA. Ranbaxy is one of the largest Indian generics producers, with a substantial share of its exports. The FDA 'has no evidence of harm to any patients who have taken drugs made in these two facilities' [44]. Nonetheless, it issued Warning Letters and a further letter identifying deviations from US cGMP requirements, such as possible cross-contamination of drug production, and of sterile processing arrangements observed during visits by two investigators, or in the reports of surveillance procedures [45]. In the case of Paonta Sahib, the first reported concern was that:

Written records of major equipment cleaning and use are inaccurate and do not provide assurance that persons double-checked the performance of equipment cleaning, because there is no assurance that those persons responsible for determining that work was performed were present at the time of equipment cleaning [[46]: 2].

The FDA followed up these Warning Letters with an Application Integrity Policy [AIP] letter to Ranbaxy, which charged that:

These and other findings indicate a pattern and practice of submitting untrue statements of material fact and other wrongful conduct, which raise significant questions regarding the reliability of the data and information contained in applications (pending and approved) that your firm has filed with the Agency [47].

Our point here is not whether pharmaceuticals produced by these plants were or were not dangerous or sub-standard, or were at enhanced risk of being so. Rather, the example shows that the credibility of production and record-keeping standards at Indian factories are negotiated globally. In protecting US consumers and re-establishing trust in FDA's regulatory processes there, the FDA also undermined trust in Indian producers within India. The Indian regulatory body was again portrayed as insufficiently stringent in monitoring production facilities under their jurisdiction.

The larger Indian companies are ambivalent about moves like this: on the one hand, they wish to show that they can meet the highest international standards, and to export their products to profitable overseas markets. But they suspect that their overseas competitors use regulatory bodies to hinder Indian companies' access to overseas markets. As the chief executive of an Indian generics company told us:

We won our case against GSK [GlaxoSmithKline] in 2005 on their drug for asthma and de facto we could market our product in the UK in 2006. It is now 2009 and we don't have permission. But they keep pointing out the deficiencies in the product and they keep asking us to do new things. It's a question of delay. So if you are blocking us on patents you are also blocking us on regulations. [2 November 2009]

Such companies also recognise that reports of decisions such as the denial of recognition by the FDA of Ranbaxy's products continue to undermine the credibility of all Indian producers. Thus the circulation of accusations and rumours about production standards and regulatory failures leads to lack of trust in all Indian products abroad, complementing and reinforcing a lack of trust within the country as well.

## Case study 3

### Trust in the DOTS programmes in India and Nepal

Both Nepal and India have adopted the central tenets of the Directly Observed Treatment, Short-course [DOTS] programme for their government-run TB programmes. Based on five core elements - political commitment; sputum microscopy; short-course chemotherapy, including the direct observation of treatment; an uninterrupted supply of drugs and finally, recording and reporting systems [48]. Much has been written on trust - or lack of it - in relation to the direct observation component in that there seems to be little trust of either the patient [49] or to the patient's family members [50,51] with direct observation as core component. In this case study, we broaden out the question of trust in the provision of TB services, to include the companies that provide the drugs and how this resonates with understandings of trust in the private sector.

In 1997, an estimated 50 percent of tuberculosis cases in India were treated-partly or completely-in the private sector [52]. Private physicians we interviewed in India frequently complained that the government run TB services were flawed in a number of ways. Some of these criticisms focused on the choice of government regimen itself - and that it was based on an intermittent treatment, not daily therapy; or that the categories of treatment were sub-optimal (particularly the so-called retreatment regimen, which added just one drug to the others and could further stimulate drug resistance) for example. However, criticism also focused on a general lack of trust of government services: patients would not trust free services, we were told, and patients would often rather pay for services to avoid government clinics. Issues included patient waiting times, that services are not flexible, and that the services are best only for the poor. For example, one Kolkata private practitioner said 'if a patient can afford the medicine I don't send them', echoing a general sense that the DOTS programme was really only for the poorest of patients. Lack of trust in government service was a central component of these criticisms.

Meanwhile, those workers involved in the public sector and the development and provision of DOTS services saw the availability and provision of medicines in the private sector as a particular problem for TB control. Valued at $94 m, 74 per cent of the of TB drugs sales were in the private sector in 2006 [53]. Our review of CIMS in March 2007 revealed 36 companies in India producing Rifampicin products; 19 in uncombined form and the following range of fixed dose combinations [FDCs]; 34 in combination with isoniazid; 12 with isoniazid and ethambutol; 19 with isoniazid and pyrazinamide; and 19 with the four drugs together. When we combined the data from three listings the total number of producers marketing a range of single and combined drugs went up to 52 (though in several cases, a single firm - such as Cadila and Wockhardt - markets drugs under two or even three names). The seven companies listed by IMS with the highest sales figures in 2006 were: Lupin Labs. (45.4 per cent) Macleods Pharma (19.7 per cent), Novartis (6.5 per cent), Shreya Lifescience (4.9 per cent), Cadila Pharma (4.7 per cent), Concept Pharma (4.5 per cent), Wockhardt (2.5 per cent), Themis Medicare (2.2 per cent). Each produces a different range of FDCs that feed into this intensely competitive local market. This wide availability of combinations might contribute to confusion about the currently-regarded appropriate treatment regimes. In 1989, in a study of Mumbai slums, researchers found that 100 private practitioners had prescribed 80 different - mostly inappropriate and expensive - drug regimens to their patients with pulmonary tuberculosis [51]. In Lucknow in 2007, a survey of 141 physicians treating TB patients found large proportions (up to 70 per cent for some items) either over-prescribing or under-prescribing anti-tuberculosis drugs, with private practitioners wrong more often than those in the public sector [54].

This situation was described as 'therapeutic anarchy' by one WHO official we interviewed. He saw the existence of many drugs of unknown quality as a major obstacle to controlling TB, and as a major concern for the rise of resistant strains of TB [MDR-TB] [see also [55]]. However, public health practitioners tended to characterise the bigger companies - particularly Lupin - as behaving more ethically, and being more trustworthy than the smaller ones. They blamed a range of practitioners, from village doctors and 'quacks', to the medical representatives of smaller companies promoting their products unethically. In short, they suspected the commercial interests of smaller companies and those of medical practitioners, and this confirmed for them the need for centralised policies and regimens.

The implications of these trust related issues - not around patients, but of companies and their products - remain uncertain. Most government programmes now procure their drugs internationally, from WHO pre-qualified sources. Thus the India and Nepal national tuberculosis programmes procure their drugs, not directly from the drug companies, but through the Global Drug Facility (GDF) procurement mechanism, housed by the WHO. It is a mechanism to 'promote standardization' and 'offer assured high-quality TB drugs and cheaper prices'. Its 'mandate is to contribute to the realization of the TB-related Millennium Development Goals and to the eventual elimination of TB through the provision of timely, quality assured and affordable anti-TB medicines and related supplies' [56]. Publicly procured drugs in both India and Nepal are provided via this mechanism, and the companies supplying this are dominated by Indian companies and include Lupin, Cadila, and McLeods. DOTS programmes cannot buy their drugs directly from companies, because funding of the buying of drugs is linked to the Global Fund [GF] policy. The GDF is 'a trusted partner for such major funding bodies as the Global Fund to Fight AIDS, Tuberculosis and Malaria' [57]. In short, countries who want to purchase their drugs using GF money have to use this source.

Thus even though national programmes order their drugs increasingly through internationally trusted sources, the lack of trust of government services remains a problem (but is rather unrelated to drug procurement and distribution). Drug availability in DOTS is high in both India and Nepal, but the quality of services, waiting time, closing hours, convenience, stigma etc are the major barriers to wider use of Government DOTS facilities. In India, while three quarters of the sales of TB drugs by value remain in the private sector, not the national programme, the problems of the wide range of treatments provided by individual practitioner will remain - and will plausibly contribute to more opportunities for MDR-TB to spread. Both sectors, then, contribute to the unmet need for appropriate treatment. How does trust come in? More trust between the public sector and the private sector might improve the quality of private sector provisions both through regulation and in-service training. More trust between patients and government services might reduce costs to patients, provide more satisfactory care, and more success in getting patients to follow drug regimens fully. Although considerable efforts have been made by WHO and others to improve quality control and raise trust in TB drugs, these efforts address only part of the problem, which inheres in the low levels of trust by patients in public sector services in general, and the inability of the public sector to educate and engage private practitioners successfully.

## Conclusion

In the sphere of medicine and public health, trust is crucial to the successful meeting of important goals - but current literature focuses too much on trust in patient-practitioner relationships, and fails to acknowledge the role of trust in other sectors. Pharmaceuticals production, distribution, retail and prescription processes are part of complex global assemblages, and so issues of trust permeate not just local and national settings, but also must be understood in their global contexts. While in some parts of these assemblages, trust relationships work in 'disembedded' ways, detached from local contexts and personal relations and involving generalised trust, in other parts of the same systems - sometimes involving the same people - trust may be based on exactly those personal networks and relationships that require face-to-face contact in local contexts. While these different kinds of trust relationships sometimes work in synergy (as Giddens argues) [7], as often as not they work against each other.

Lack of trust generates high transaction costs, and in this paper we have specified the general outlines of some of those costs - for the health system as a whole, and for patients in particular - in the pharmaceuticals markets of India and Nepal. By looking beyond trust in patient-practitioner relationships - which can too easily lead to a 'blaming of the victim' (or at least a blaming of the patient who does not follow the practitioner's recommendations) - we have cast a wider net. We suggest that issues of mistrust characterise many (if not all) relationships amongst stakeholders engaged in South Asia's pharmaceuticals supply chains. The presence of competing stake-holders - each of which feels justified in pursuing its own interests - undermines trust either within the same or other sections of the health sector. Each tends to argue that if others would only trust them, costs (economic, political social or health costs) would fall. But the failure to achieve some coherence in attempts to reform the situation points to wider, systemic, problems. Governance reform is not so much about 'implementing designs created by committees of technocrats. Rather, the first order of business is to restore credibility to the state itself' [58]. The inability of the myriad commissions of enquiry appointed by the Government of India to review pharmaceuticals since 1996 is testimony to the absence of sufficient legitimacy within the state to achieve such a goal [59], as is the failure of the Nepal Government to see through its pharmacy reform measures. Building trust - in a health system as a whole - is a complex operation, not helped by international interventions that (at one level) seem to be about building trust but at other levels work to undermine it. Where the public health sector is failing as badly as in India and Nepal, trust and mistrust have become personalised: motives are continually suspect; one set of actors rarely praises the trustworthiness of others; change is resisted tenaciously. And 'who will regulate the regulators themselves' remains a serious problem.

## Competing interests

The authors declare that they have no competing interests.

## Authors' contributions

PB carried out the desk research in Edinburgh on regulatory practices and helped to draft the manuscript. IH was responsible for the field research in Nepal and helped to draft the manuscript. RJ, IH and others conceived of the study, RJ was the PI, and participated in its design and coordination, in data collection, and helped draft the manuscript. NR and MS were partners in the data collection in Nepal. SMR was a partner in the data collection and analysis of material from India. All authors read and approved the final manuscript.

## End Notes

^1 ^See, for example, the scandal over the recognition of new medical colleges by the MCI, whose Chair Dr Ketan Desai was forced to resign in April 2010. The Indian Central Bureau of Investigation requested 'specific complaints of money demanded by MCI officials for giving approval/permission to colleges without requisite infrastructure or other things' and had received 154 such complaints in the following month (http://beta.thehindu.com/news/article434610.ece). In March 2010, the MCI asked the Government to penalise drug companies who offer doctors payments or other benefits (http://www.pharmainfo.net/og/pharmindia/mci-asks-health-ministry-make-pharma-cos-punishable-gifting-docs)

^2 ^For more details see the website http://www.csas.ed.ac.uk/scaffolding/new_page_root/research_projects/tracing_phramaceuticals.

^3 ^Producers or their medical representatives offer retailers additional boxes if retailers bought their product. In some cases these were 'buy one, get one free' but sometimes were more generous.

^4 ^The Pharmaceuticals Security Institute [PSI], one source of this claim, is linked to the International Federation of Pharmaceutical Manufacturers & Associations, whose Director-General serves as the PSI President.

^5 ^This study used flawed methods of collecting samples, and therefore cannot be relied on, but the contrast with the industry estimates is too large to be ignored.
